# Male Victims’ Experiences With and Perceptions of the Criminal Justice Response to Intimate Partner Abuse

**DOI:** 10.1177/08862605211001476

**Published:** 2021-03-23

**Authors:** Eugene Emeka Dim, Alexandra Lysova

**Affiliations:** 1 University of Saskatchewan, Saskatoon, Canada; 2 Simon Fraser University, Burnaby, British Columbia, Canada

**Keywords:** Canada, intimate partner abuse, male victims, criminal justice, administrative abuse, qualitative study

## Abstract

Intimate partner abuse (IPA) carries severe physical and psychological consequences for victims, and the police and courts are some of the essential formal structures that help victims address their victimization. Studies suggest that male victims of IPA are reluctant to speak about or report their victimization to the police. This qualitative study examines the experiences male victims of IPA had with the criminal justice system (i.e., the courts and police). We conducted interviews with 16 men who had experienced IPA in their previous relationship in Canada. Two major themes about the police response were identified: the barriers to contacting the police for help and negative experiences with the police response. We found that men who chose not to contact the police did it due to the negative expectations of being ridiculed by the police, not being believed, and fear of being arrested. Those men who called the police for help reported unfriendly and antagonistic police treatment and the police’s reluctance to charge abusive female partners. The themes that reflected the male victims’ interactions with the court pointed to: (1) legal and administrative abuse by female partners, including false accusations and manipulations of child custody, and (2) a general bias against men in the courtroom. This study brings attention to the need for law enforcement officers to be aware of the experiences and perceptions male victims have of the criminal justice system and the need for the criminal justice system to create more inclusive strategies to help male victims of IPA.

## Introduction

The World Health Organization (WHO) defines intimate partner abuse (IPA) as behavior within an intimate relationship that causes physical, sexual, or psychological harm, which includes acts of physical aggression, sexual coercion (or forced sexual penetration), psychological abuse, and controlling behaviors between intimate partners (Krug et al., 2002). IPA is a pervasive problem that carries physical and psychological consequences for its victims (Ansara & Hindin, 2011). Some of these consequences include injuries, severe pains, and mental health problems (Campbell, 2002; Dichter & Rhodes, 2011; Straus et al., 2009).

Since IPA research began, studies based on a set of premises known as a gender paradigm (Dutton & Nicholls, 2005) have conceptualized IPA mainly as violence perpetrated by men against women. However, since the 1980s, empirical studies have also shown that the majority of IPA is bidirectional (Langhinrichsen-Rohling et al., 2012: Melander et al., 2010; Whitaker et al., 2007), and the prevalence of IPA among male victims tend to be similar to that of female victims (Costa et al., 2016; Desmarais et al., 2012; Park & Kim, 2017). According to the Centre for Disease Control and Prevention (CDC), about 8% of men experienced contact sexual violence, and 31% experienced physical violence (including 14.9% of severe physical violence) (Smith et al., 2018, p. 9). Despite high rates of victimization, men are reluctant to seek help, even when they need it (Addis & Mahalik, 2003). Not only are men less likely to report their victimization to the police, but they are also more likely to be dissatisfied with police intervention than female victims of IPA (Breiding et al., 2014; Burczycka, 2016). This study explored the experiences of male victims of IPA with the criminal justice system—primarily the police and court—in Canada. This qualitative study aimed to get a contextualized understanding of male victims’ experiences with IPA and the challenges they face when they deal with the criminal justice system’s response to their IPA victimization.

## Prior Research

### Male Victims and Police Response

For the most part, victims of IPA seek the help of the police as a means of formal support in escaping their victimization. A meta-analysis by Sarkar (2008) found that women who contact the police have a 59% reduced risk of intimate partner sexual assault. However, the results tend to be different when male victims seek police intervention for their victimization. A study from the U.S. National Crime Victimization Survey revealed that arresting a perpetrator significantly reduced the odds of female revictimization by 45% but had no significant effect on reducing male revictimization, suggesting a lack of deterrent effect of arrest on female perpetrators (Cho & Wilke, 2010).

Moreover, a review of seven studies by Felson (2008) showed that offenders who assaulted women were more likely to suffer legal consequences than those who assaulted men, whether their victim was their partner or someone else. In a study of 302 men seeking help for IPA victimization in the United States, the police and domestic violence (DV) agencies were reported among the least helpful support services (Douglas & Hines, 2011). In another study, male victims of IPA were more likely to complain about a lack of investigation regarding their victimization (Felson & Paré, 2007). Male victims had also reported that, when they called the police during an incident of female-perpetrated violence, the police did not always respond or take a report (Cook, 2009; Hines & Douglas, 2009). Another study by Buzawa and Hotaling (2006) found that law enforcement officers were significantly less likely to give male victims information about available services, including restraining orders, than female victims.

In some cases, the police showed no empathy or any willingness to listen to the experiences of male victims of IPA (Lysova et al., 2020, 2022; McCarrick et al., 2016). In a study of 372 male victims of IPA in the Netherlands, less than 32% of the men had approached the police about their victimization, while only 15% of the men registered an official report to the police (Drijber et al., 2013). The main reason for not reporting the abuse incident was the belief that the police would do nothing. Besides, men’s underreporting to the police can be attributed to the fear of being charged when countercharges are made against them (George, 1994). In a qualitative study of the help-seeking experiences of 38 abused men within the criminal justice system in Australia, Canada, the United Kingdom, and the United States, when the men called the police, the police arrived only in 27% of these cases and showed hostility and bias against men; in three cases out of five when the police made an arrest, the men were arrested (Lysova et al., 2020).

Within the context of these findings, a more nuanced understanding of why male victims of IPA choose to report (or not to report) their victimization to the police becomes imperative. Given the paucity of studies documenting the context in which male victims seek help from the police, this qualitative study will give a voice to the lived experience of male victims of IPA with the police and allow the police officers and other service providers to gain deeper insight into the distinct needs of this hard-to-reach population of victims (Douglas et al., 2018). Specifically, it will examine the beliefs and ideas that shape the male victim’s resistance to speak or report their abusive experiences to the police.

### Male Victims and the Courts

Both the provision of restraining orders for abuse and perpetrators’ convictions underscore the importance of the courts in response to IPA victimization. A few studies have inquired into the experiences of male victims of IPA as plaintiffs in the courts. For example, a study in Massachusetts, United States, revealed despite the gender-neutral language of the abuse prevention law, male victims were not afforded the same protections as their female counterparts (Basile, 2005). This inequality in court response occurred even though their opposite gender defendants similarly victimized male and female plaintiffs. Additionally, none of the men in the study population were able to secure custody of their minor children for more than a few days (Basile, 2005). Another study of 157 petitions involving intimate partners seeking temporary restraining orders (TRO) revealed that judges were 13 times more likely to grant female TROs against their male partners than male requests, controlled for the severity of abuse (Muller et al., 2009). Another study of 8,461 cases involving heterosexual IPA revealed that the suspect’s gender was found to be statistically significant concerning all four outcomes (i.e., the decision to file charges; to file as a felony; to dismiss for insufficient evidence, and to reduce felony charges to a misdemeanor or violation of probation) favoring female over male suspects in all outcomes (Rodney & Randall, 2007).

Moreover, it has been documented that female abusers sometimes use the court system to revictimize male partners. A study of 246 male callers to the U.S. Domestic Abuse Helpline for Men found that 72% of the men claimed that their abusers manipulated the court system to gain sole custody of the children or obtained an unwarranted restraining order against the victim (Hines et al., 2007). Qualitative studies of male victims of IPA found that men were not given a fair hearing in the court despite the evidence of female-perpetrated abuse, that they lost custody of their children more often, and that they faced a much higher burden of proof than female victims (Cook, 2009; Lysova et al., 2020, 2022).

Given that the court outcomes for IPA cases tend to favor female victims disproportionately, male victims may have a negative perception of the court when addressing their victimization. Men may also be reluctant or refuse to press charges as they might be afraid of losing custody of their children or not being given a fair hearing in court, based on the stories they have heard from other male plaintiffs. This qualitative study contributes to the existing literature by examining the experiences male victims of IPA have with the courts. The study seeks to complement the mainly quantitative research on male victims’ interactions with the court by examining men’s perceptions that can translate into male victims’ pursuit or non-pursuit of the courts to address their IPA victimization.

### Legal and Administrative Abuse

A few studies have drawn attention to the legal and administrative abuse of an intimate partner (Hines et al., 2015). According to Tilbrook et al. (2010), legal and administrative abuse involves a person using legitimate services in a manner that abuses the right of others. In the context of IPA, this involves individuals calling the police to arrest their intimate partner or spouse, even though no harm has been done to them. A scale of legal and administrative aggression developed by Hines et al. (2015) includes false accusations to authorities that the partner physically or sexually abused the other or the children, threats to take the children away, and threats to ruin the partner’s reputation at work and/or in the community. A study of 302 men who sustained severe IPA from their women partners within the previous year and sought help found that 67.2% reported that their partner falsely accused them of hitting or beating their partners (Hines & Douglas, 2010). Also, about 38.7% reported that their partners filed a restraining order against them under false pretenses; 48.9% said that their partners falsely accused them of physically abusing the children; and 15.4% reported that their partners falsely accused them of sexually abusing the children (Hines & Douglas, 2010).

False accusations are particularly harmful to victims of IPA when mandatory and pro-arrest policies are in place. Mandatory policies require a police officer to make an arrest (or encourage an arrest as a preferred option in the case of pro-arrest policies) if the officer finds probable cause to believe that an offense has been committed. Mandatory and pro-arrest policies were based on the belief that the police were being lenient with male assaulters who were often not arrested. However, there is no empirical support for the idea that the police treat violent husbands more leniently than other violent offenders (Felson, 2008). Conversely, a study using the U.S. National Crime Victimization Survey showed that the police were unlikely to arrest women who assaulted their male partners (Felson & Paré, 2007). Few qualitative studies of male victims of IPA suggest that some men avoid getting help because they are afraid of being wrongly accused and falsely prosecuted by the police or courts (Lysova et al., 2020). However, detailed accounts of male victims of IPA who experienced legal and administrative abuse are still limited. This qualitative study will contribute to the literature by providing further information on the nature and specific examples of this type of abuse against male victims of IPA in their interactions with the criminal justice system.

## Present Study

There has been a paucity of qualitative studies to date that have examined male victims’ experiences with the criminal justice system (Lysova et al., 2020, 2022; Machado et al. 2017; McCarrick et al., 2016). This study contributes to the literature by focusing on the narratives that describe the male victims’ experiences with the police and the court system to address their victimization. Specifically, we examined (1) male victims’ perceptions of the criminal justice system’s helpfulness that create barriers to contacting the police and courts, and (2) the quality of interactions with the police and courts among the men who contacted the criminal justice system to get help for their IPA victimization.

## Method

### Procedure

The invitation to participate in this study was advertised in several non-governmental organizations that provided help and assistance for men and boys. The inclusion criterion for the study was the experience of IPA among men above the ages of 18 years in past intimate relationships. The intimate relationships considered for this study included married, common-law, and dating relationships. We used a convenience sample in this study. Men who were interested in participating in this study called the first author to arrange the time for the interview. Participant consent was obtained either orally or in writing before any interviews commenced. A compensation of $20 was offered for participating in this study. However, all but one of the participants declined the remuneration. The respondents insisted that they did not want to do the interviews for money but wanted to tell their stories. The University Ethics Committee approved this study. Thirteen interviews were conducted via the telephone, while three of the male participants requested to respond to the interview questions via writing due to the traumatic nature of the subject matter. The telephone interviews were recorded with the permission and consent of the respondents. Data collection lasted between October and December 2016, and analysis of the transcripts took place between January and March 2017.

The 16 male participants were asked broad, open-ended questions that allowed the men to present their experiences without being led by the interviewer. During the interviews, participants were asked, among other questions, about reporting the abuse incidents to the police (i.e., Did you report the abuse incident to the police? What were your reasons for reporting or not reporting it to the police? Have you heard of any cases where male victims reported the abuse incidents to the police and what was the outcome of these reports?) and about their interaction with the courts (e.g., What was your experience with the courts for your abuse incident?). Each interview lasted about one hour.

### Participants

The participants in this study were 16 men who experienced IPA in heterosexual relationships and agreed to participate in this study. Table 1 outlines some of the socio-demographic characteristics of the participants in the study. The men ranged in age from 34 to 75 years (Mean = 57; *SD* = 11.46). Most of the respondents experienced both physical and psychological abuse from their female partners. The years of living with the ex-partners (i.e., the period of events) ranged from 1 to 27 years (Mean = 8.7; *SD* = 7.45). At the time of the interview, most of the men were single and employed. Table 1 also indicates whether each of the participants interacted (yes or no) with the police and court as part of their help-seeking behavior or if their partners or a third party called the police. Eight of the men interacted with the police, and ten men had contact with the court. Only five men interacted with both institutions. In this sample, three men had not come to contact with either police or court.

**Table 1.** Socio-demographic Characteristics of Male Participants and Their Police and Court Contact (*n* = 16).

Pseudonym	Age	Occupation	P.V. Type^a^	Current Marital Status	Period of Event	Police Contact^b^	Court Contact^b^
A	48	Musician	PA & PsyA	Single	2011–2012	No	No
B	56	Self-employed	PA	Single	2011–2015	Yes	Yes
C	70+	Self-employed	PA	Single	1990–1997	No	No
D	47	Business analyst	PA & PsyA	Single (Dating)	2009–2015	No	Yes
E	73	Retired Psychologist	PA & PsyA	Married (now)	1969–1991	No	No
F	41	Government worker	PA & PsyA	Single	2006–2014	Yes	No
G	49	Accountant	PA & PsyA	Single	2003–2008	Yes	Yes
H	34	Student	PA & PsyA	Single	2014–2015	Yes	Yes
I	50	Management consultant	PA & PsyA	Single	2012–2015	Yes	No
J	58	Aircraft Technician (RCAF)	PA & PsyA	Married	2006–2007	No	Yes
K	60+		PsyA	Single	1983–2007	Yes	No
L	57	Writer	PA & PsyA	Single	2008–2011	No	Yes
M	73	Retired	PA & PsyA	Single	1985–2008 & 2012–2015	Yes	Yes
N	59	Government worker	PA & PsyA	Dating	1985–1996	Yes	Yes
O	75	Criminal Lawyer	PA & PsyA	Single	1969–1985 &1989–1999	No	Yes
P	59	School bus driver	PsyA	Single	2005–2013	No	Yes

*Note*. ^a^PA: Physical abuse; PsyA: Psychological abuse.

^b^The contact with the police and/or courts includes a respondent’s any interaction with these institutions due to their own help seeking or when the respondents’ partners or a third party contacted the institutions.

### Data Analysis

Thematic analysis was applied to the data to report the experiences, meanings, and reality of the respondents (Braun & Clarke, 2006). The generation of themes for this study followed the inductive approach that contained no theoretical preconceptions and reflected the men’s interpretation of their own experience of reality (Braun & Clarke, 2006). The first phase of the thematic analysis involved reading the transcripts to ensure familiarity with the narratives and develop initial approaches for codes. Then, based on the recurrences within the data, which reflected common issues to the participants, the authors developed the codes. Codes that had similar and substantial content were merged to form a final set of codes. Then the analysts grouped those initial codes into a smaller number of themes and worked on identifying the subthemes that emerged within each theme. After checking the themes back against the dataset by going through the transcripts, the authors resolving any alternative ideas for codes and gave themes and subthemes meaningful labels. To ensure the trustworthiness and credibility of the results, we reviewed and confirmed our qualitative study to follow the COREQ checklist (Tong et al., 2007). Reflexivity was applied throughout the study, especially in the sampling and final editing process. This process was mainly achieved by reflecting on the authors’ gender and theoretical positions and identifying gaps in the study. Given a small number of the participants in this study, the frequency of responses for each theme is not provided.

## Results

Four themes were developed, that is, two for the experiences with the police response (the barriers to contacting the police for help and negative experiences with the police response) and two for the interactions with the courts (female’s legal and administrative abuse in the courtroom and “automatically guilty by the letter of the law”). Each of the themes and subthemes is described with supporting quotations below.

**Table 2.** Themes and Subthemes Within Men’s Experiences With the Criminal Justice System.

Theme	Subtheme
**Experiences with the police response**	
1. The barriers to contacting the police for help	1. “You can’t stand up to a woman, what’s the matter with you?”
	2. “The police would not believe me”
	3. “I knew they would take me away”
2. Negative experiences with the police response	1. “The police was completely against me”
	2. “Not enough evidence”
**Interactions with the courts**	
3. Female’s legal and administrative abuse in the courtroom	1. “I had no doubt she was setting me up.…”
	2. Manipulating the child custody
4. “Automatically guilty by the letter of the law”	

### Experiences with the Police Response

Some men in our sample had firsthand experiences with police intervention in their abuse cases, while others perceived barriers to informing the police of their victimization. Hence, two major themes were identified, that is, the barriers to contacting the police for help and actual negative experiences with the police.

#### Theme 1: The barriers to contacting the police for help.

This theme outlines the men’s major negative perceptions of the police that stopped them from contacting the police in cases of their IPA victimization. The analysis identified three subthemes, that is, the anticipated negative treatment of male victims of IPA by the police (“you can’t stand up to a woman, what’s the matter with you?”), male victims not being believed (“the police would not believe me”), and fear of getting arrested (“I knew they would take me away”).

**Subtheme 1: “You can’t stand up to a woman, what’s the matter with you?”** Some men in the study had a negative perception of the police because they believed that the police would either not care about their abuse or shame them for their victimization. Due to the expectation of a negative response, these men never called the police to their abuse incidents. For example, Participant J revealed:

I never called the police and in retrospect, I don’t think calling the police would have made any difference at all. I didn’t call the police partly because of shame.… They may say: “A man like you can’t stand up to a woman, what’s the matter with you?”

The social stigma seemed to have dissuaded this participant and some other participants from considering police intervention for their victimization. Another Participant (A) did not call the police for help because “the police hold the narrative that only men are abusers,” and Participant E. echoed this: “I worked in the Custody and Access field. The police and the courts sided with the women’s stories.” Participant O, a lawyer who observed the criminal justice system’s bias against male victims in his professional life, also never reported his own wife’s physical abuse to the police.

**Subtheme 2: “The police would not believe me.”** Another recurring theme among the participants was the impression that the police would not believe their reported victimization. Participant J did not contact the police because he assumed that “the police will not believe me.” Other men elaborated on this and attributed the police’s expected non-intervention to the non-physical nature of the aggression the men experienced. For example, Participant E shared: “Her abuse was verbal and only destroyed my mind. There were no physical marks.” Respondent P echoed this concern: “The police would not believe me, and it was verbal, and it was like they would do nothing.” Male victims referred to the difficulties for a victim of psychological abuse to prove their victimization, as such forms of abuse do not leave visible scars or injuries on the victim (Drijber et al., 2013). For example, Participant K reported:

[Psychological abuse] is much more difficult and more nuanced [than physical abuse], which is probably more of the problem to prove the psychological harm of any victim. It takes a lot more effort, a lot more work, a lot more investigation, and much more time to show what emotional harms were.

Some men reported that their abusive female partners did not realize that their behavior was abusive, while some men blamed themselves for being blind to the psychological abuse they experienced. For example, Participant I shared:

I just didn’t see it, I had minimized it, I had bought into her narrative that it was my fault and that I was working very hard to change myself to do something different, to not do things, to do more of other things, to change so that I wasn’t causing her to be angry and upset. So, I really bought into the fact that it wasn’t violence, it wasn’t abuse, it was just me not being up to the tasks, of not being a good relationship partner.

Participant K echoed the experiences of other men:

Well, in retrospect it is clear, [but] at that time it wasn’t clear at all. I had to learn about these things [psychological abuse] and to better understand what the effects were and what she was doing, and I don’t say that she was doing this intentionally and I am not sure she understood what she was actually doing, but that doesn’t change the circumstance.

The lack of evidence for psychological abuse and the realization that the perpetrator is oblivious to their abuse could have caused male victims to be less confident about reporting their victimization to the authorities. The nature of violence may also be deemed too ‘little’ by some male victims to seek police intervention. The participants also felt that they could handle this type of abuse, and they found no need for external intervention for their victimization. As Participant K put it: “I didn’t think that it was a police matter. I had no clue about how police intervene in domestic situations. It was something entirely foreign to me.” Participant P explained his reluctance to call the police in case of psychological abuse:

Because they would not believe me and it was verbal and it was like they would do nothing, I know it and that was the right thing to do. I knew I had to get out and I had to take care of it myself and that was what I did.

**Subtheme 3: “I knew they would take me away.”** Some men who never contacted the police for help pointed to the possibility that their female partners might abuse legal and administrative institutions for their benefit. The legal and administrative abuse sums up some of the participants’ negative perceptions of the police. For example, Participant J reported that, “…there was a certain amount of fear that if I went to report her, I will be the one who gets arrested.” In fact, this participant was later arrested for defending himself from his ex-partner’s physical attacks. Respondent E echoed this concern:

During the abusive situation, I didn’t report it because I knew they would just take me away. I knew she would lie, and she is very good at just suddenly falling down into tears and being the most sympathetic person, she could just go from one extreme to the other and I knew that she would do that when the police come and I would be toast, I just knew it.

Participant K was in fact arrested five times while he claimed he was the victim of abuse. Another Participant (G) reported he was falsely accused by his ex-wife and arrested for abuse he did not perpetrate:

A couple years after we split up, she came into my home to pick up the kids and she had come a few hours early, and I told her to wait because the kids were still asleep. She got angry and was violent to me and my son. And 911 was called. Afterwards the police arrested me, because she accused me of violence against her. But because I had witnesses there, when it finally went to court over a year later, it was thrown away by the prosecution, because they realized that a totally different thing had happened than what she had stated to the police officer.

#### Theme 2: Negative experiences with the police response.

Some of the participants spoke about the unsatisfactory and negative outcomes they experienced when they sought police intervention. Two subthemes specified unhelpful and antagonistic police treatment of the men.

**Subtheme 1: “The police were completely against me.”** This subtheme specifies the situations when the abused men who called the police were denied attention and help. For example, Participant G reported this incident:

I called the police once after the incidence that she pushed me and hit me and then got mad and locked herself in the bedroom, and I called the police and complained about it, and they said, “Is anybody in immediate danger?” and I said, “No” and they said, “Well, there’s really no reason for us to come out.” And that was the end of it and at that point I just said the police were useless.

Another Participant (M) reported that he was not only attacked with an object and physically assaulted in front of the police but was also ridiculed by the police officers while being attacked. Another Participant H reported that his female partner physically abused him in the presence of two police officers who just laughed at him:

A lot of throwing things, she hit me a few times, she punched me in the face a couple of times, she punched me right in the ribs in front of two police officers, and on the way out they laughed and called me her punching bag.

**Subtheme 2: “Not enough evidence.”** Some of the respondents reported that the evidence of their abuse could not be used to press charges against their ex-partners. For example, Participant M revealed:

I phoned the police four times. They would come, they would tell her no contact, they would give us a piece of paper no contact, she had to go and live in a Motel. Within two, three days, she was back home, and charges were dropped. The last time this happened, even my lawyer was asking, how come the police drop the charges every time and you are still in the hospital and they are saying “not enough evidence’ and you are lying in the hospital with broken ribs and heart attack.

Respondent I shared that the police officers he called to get help discouraged him from filling out the report. Moreover, when he told them what happened,

The police officers said, “What did you do to cause that?” and I said, “I didn’t do anything” and they said, “Come on, that can’t be possible, you had to have done something to cause this, what did you do to her? How did you instigate this?” and they just kept going on and on and literally it took me about half an hour of just continuing to advocate on my own behalf.

The negative experiences with police responses the men reported informed their negative perception and lack of confidence in the police, who they felt could not relate with their victimization.

### Interactions With the Courts

Most of the men in this study who had first-hand experience with the court, for example, child custody cases or hearing for abuse incidents, responded about their experiences with and perceptions of the DV courts. Two major themes were found, that is, female’s legal and administrative abuse in the courtroom and a more general bias against men in the courtroom—“automatically guilty by the letter of the law.”

#### Theme 3: Female’s legal and administrative abuse in the courtroom.

This theme details major concerns the men had about their abusive female partners using the court system against them. The subthemes found in this theme include false or abusive testimonies by their female partners in court and manipulated child custody.

**Subtheme 1: “I Had No Doubt She Was Setting Me Up.…”** Some of the respondents noted that their female partners were abusive in the relationship and then falsely accused male partners and misrepresented their own involvement in violence in court, which impacted the nature of judgment in these cases. For example, Participant B noted that his female partner was abusive towards himself and their child:

The violence was coming from her not wanting to wake up and feed this baby and when she would finally move, she was with rage and anger and she handled the baby very rough.… She would assault me when the baby was either in my arms or in her arms at the time this was going on.

However, when Participant B contacted the police and was assured that his wife would be arrested for the serious abuse she committed, the police “didn’t arrest her, they came to the house and within five minutes they took her and the baby to the women’s shelter and then she instantly became a victim at the women’s shelter.” Then in court she false accused her husband of abusing her and Participant B lost custody of his son:

She came to court with the affidavits with ridiculous stories of how I punched her in the ear every time she was breastfeeding and kept her locked at home.… The current court order is that I get to see my son two times a week six hours a piece and I have to pay $150 per month for maintenance.

Another Participant (L) reported that his ex-partner was verbally abusive in court and the judge described her as “rigid and frustrated,” but the male plaintiff lost his case. It is also possible that stories of the negative outcomes for male victims can create a negative perception of the courts and discourage other men from seeking redress.

**Subtheme 2: Manipulating the child custody.** Some men in our sample reported concerns over losing their child custody cases. For example, Participant L, despite his partner admitting that she was physically abusive to him, lost custody of his daughter. His female partner used Participant L’s health condition to declare him as an unfit parent (he was diagnosed with a fistula as he had large parts of his large intestine removed and had a bag on the side for his bowels). The female abusers utilized the perception of the gendered-aggressor narrative to gain full child custody. Participant B regretted that he had reported his incident of IPA victimization to the police. He shared that after he had suffered a “bad assault” that left him bleeding in three places,

[T]he counselor at child protection has told me, “Well, you have to go to the City Police, and then you go to the courthouse and you file for custody.” So I went to the City Police and they are telling me that this is serious and then they tell me that they’re going to arrest my wife and they said, “Don’t worry, we are not going to put her in jail, we are going to arrest her, for this is criminal charges and she’ll have to sign a promise to appear and then she’ll go to court and hopefully the judge will be understanding and she’ll have to get counseling or something like that.”… Well, they didn’t arrest her, they came to the house and within five minutes they took her and the baby to the women’s shelter and then she instantly became a victim.

#### Theme 4: “Automatically guilty by the letter of the law.”

This last theme underscores the lack of confidence male victims have with the courts and the process through which their cases were addressed in the court. Participant J noted how he felt the court would be biased against him and the possible impact of such bias:

I found out from my lawyer that basically, by the letter of the law, if a man is present and there is an incident, the man is automatically guilty and automatically arrested. She said that the police don’t have jurisdiction on that, they have to arrest you. So even if I reported it, I probably would have been arrested anyway. In this regard, I have no confidence in the justice system.

At the same time, there was one positive outcome for Participant N who managed to get custody of his daughter through the court system. However, he noted that his lawyer had to maneuver through the court proceedings before they got a favorable judge for their case. According to Participant N:

My lawyer used to check in the morning, to see who the judge was and if the judge was very pro-women, he would call in sick because he knew he would lose in court and it is totally unfair, and from that perspective, even though what he was doing was probably wrong, it just sort of protected me from the bias in the court system.

## Discussion

This qualitative study sought to investigate interactions the male victims of IPV in our study had with the police and courts due to their abuse and the perceptions of those men who avoided contact with the criminal justice institutions. This study’s findings are consistent with other studies that point to the negative outcomes male victims face from the police and the court system (Cook, 2009; Lysova et al., 2020, 2022; Rodney & Randall, 2007). In terms of reporting the abuse to the police (Theme 1), male victims were reluctant to do so for fear of being seen as the perpetrator rather than the victim of IPA. Those who reported the abuse to the police faced unhelpful and antagonistic police treatment that questioned men’s reports of victimization (Theme 2). Some previous studies found that the police and victim services tend to view male victims of IPA as perpetrators (Douglas & Hines, 2011; Lysova et al., 2020, 2022), and this fact aligns with the fear the male participants expressed in our study (first and second subthemes of Theme 1). Moreover, calling the police in the incidents of IPA can indeed put the male victims at risk for being arrested (third subtheme of Theme 1) (Cook, 2009; Douglas & Hines, 2011; Kelly, 2003; Lysova et al., 2020). Several studies found that male victims reporting their victimization to the police sometimes got arrested instead of the female perpetrator (Lysova et al., 2020; Stitt & Macklin, 1995).

The use of pro-arrest or mandatory arrest policy in IPA incidents may lead the police, guided by the gender paradigm that tends to take the domestic abuse of women more seriously than that of men, to a revictimization experience for the male victim. Although the pro-charging policies adopted in Canada during the 1980s have significantly contributed to the strengthening of the criminal justice system’s response to spousal abuse (Canadian Department of Justice, 2017), these policies could potentially cause male victims to have a negative perception of the police and discourage them from reporting their victimization to the police. The gender paradigm, a view that men are predominantly the perpetrators of IPA against women (Dutton & Nicholls, 2005), tends to shape the discretion of the police to disregard male victims, while the legal and political systems tend to be designed to mostly respond to the abuse of women (Hines & Douglas, 2009; Kelly, 2003; Tsui et al., 2010). This context may explain why male victims of IPA in this study were reluctant to admit and report their victimization to the police for assistance. These findings align with the 2014 GSS (victimization) survey report, which revealed that male victims were less likely to report their victimization to the police than female victims (Burczycka, 2017). Besides, men were more likely than women to report being very dissatisfied with how the police handled their situation (Burczycka, 2017). The disparities in reporting to the police tend to be reflected in reports on spousal violence from police-data sources that show that the majority of victims are women.

One possible explanation suggested for the different reporting rates is the less serious nature of violence experienced by male victims (Canadian Department of Justice, 2017). However, an analysis of the 1999 and 2014 GSS (victimization) data revealed that men were similarly or more likely to suffer severe violence than female victims (LaRoche, 2005; Lysova et al., 2019). Specifically, among victims of physical and/or sexual intimate partner violence in the ongoing relationship, as measured by the 2014 GSS, 22% of men and 19% of women experienced the most severe forms of physical violence along with high controlling abuse (Lysova et al., 2019). A study of help-seeking among male victims of IPA in Canada based on the 2009 and 2014 GSS found that unemployed men and those residing with small children were less likely to use formal help (including contacting the criminal justice system) for their abuse than informal help (Lysova & Dim, 2022). These findings suggest that the most vulnerable male victims of IPA may be particularly deterred from contacting the police and seeking other formal help due to fears to lose custody of their children and/or being arrested.

Noteworthy, that this study did not detect any substantial differences between the help-seeking experiences of men who reported about the abusive incidents that occurred recently and more than 20 years ago. It appears that, while pro-arrest policies in relation to IPA seem to exacerbate male victims’ experiences with the criminal justice system, the treatment of male victims of IPA was not much better before the policy reform in the 1980s (Dutton & White, 2013; Lysova & Dim, 2022). This points out to social perception of masculinity and stigma as one of the pervasive male victim’s barriers to seeking help (first subtheme of Theme 1) (Addis & Mahalik, 2003). A male victim’s perception that his masculinity may be challenged can be one of the reasons why he refuses to report his victimization. Sometimes, male victims are reluctant to report their victimization to the police, even in the most severe cases due to the prevailing norms regarding masculinity (Lysova et al., 2022; Muller et al., 2009). These norms may cause male victims to be reluctant to express fear or sad emotions or call the police even when they have good reasons to do so (Addis & Mahalik, 2003; Hines et al. 2007). Male victims may avoid expressing their victimization to others in order to seek personal avenues of addressing their victimization without necessarily being in the public eye.

The victims may also feel that seeking police intervention may not bring an end to their victimization. Furthermore, Ansara and Hindin (2010) found that male victims were less likely than women to disclose their victimization or seek assistance if the violence was relatively minor or less severe. Additionally, male victims may perceive their victimization as minor, even if such abuse falls under the category of severe violence or high controlling behavior (Dixon et al., 2022).

Concerning the treatment of male victims in the courts, respondents reported how their female partners engaged in legal and administrative abuse, including making false allegations (Theme 3), and these cases are similar to the findings in other qualitative studies (Cook, 2009; Lysova et al., 2020; Morgan & Wells, 2016; Tilbrook et al., 2010) and also quantitative studies (Hines et al., 2015). It was concerning to hear about men’s experiences with losing custody of their children resulting from the female partners’ use of legal and administrative abuse (second subtheme of Theme 3). A study of families experiencing parental separation revealed that 76.5% of the child’s custody was given to the mother compared to 6.5% given to the father (Juby et al., 2005). This fact could influence how men and women in child custody and divorce proceedings may perceive their chances of success.

The negative experiences with and perceptions of the courts expressed by some participants in this study have been supported by some previous quantitative studies (Basile, 2005; Muller et al., 2009). For some of the respondents, the concern is that gender-neutral laws produced outcomes that rarely favor the male plaintiff (Theme 4). Such experiences and perceptions were enough to make them lose confidence in the courts’ ability to address their victimization. It is also possible that the negative perceptions male victims have of the courts may be different from the reality if they went to court and found a helpful response. However, such perceptions of negative bias may be enough to dissuade them from seeking redress from the court system. This limits the range of possible helping services male victims seek, especially from formal sources. A report from the 2014 GSS on victimization revealed that male victims were far less likely to report having a restraining order enacted against their current or former spouse than female victims, and this finding is similar to the 2009 GSS data (Burczycka, 2017). The data also reported that informal support, that is, family members, in the form of help most often sought by male victims (Burczycka, 2017; Lysova & Dim, 2022).

### Limitations of the Study

One major limitation of this study is the retrospective nature of the study as the participants had to recall their experiences of IPA from previous relationships, some of which occurred years ago. Such research can be affected by cognitive bias, and memories of such events may have been altered (McCarrick et al., 2016). However, these recollections of the abusive events are still very valuable and need to be heard, especially in relation to such a hard-to-reach population as male victims of IPA (Douglas et al., 2018). Secondly, the age group of the participants interviewed was between 35 and 75 years, which does not capture the sample of younger men who are more likely to experience IPA than older men (Statistics Canada, 2016: 36–38). Older individuals may be more likely to seek outlets to express their experiences or seek help for their victimization (Barrett & Pierre, 2011). Also, this study was based on a small sample size (although appropriate for qualitative research), and there is a possibility of the self-selecting nature of the sample of respondents. This implies that men who face more barriers with the criminal justice system may be unlikely to come forward to contact and discuss their victimization experiences with the researchers. It is also important to add that our study did not focus on the IPA laws. We acknowledge that the laws have changed overtime about the year of the occurrence of the IPA events. Although our study did not detect any major differences between the help-seeking experiences of men who reported about the abusive incidents that occurred recently and more than 20 years ago, future research should examine this question.

## Conclusion

This study sought to understand the interactions of male victims of IPA with the police and court system. The study revealed the male participants’ revictimization experiences with the criminal justice system negatively affected their perception of formal help-seeking. These results suggest the need to encourage a more balanced and gender-informed perspective of IPA among criminal justice professionals and practitioners. Law enforcement officials should be more aware of the existence and experiences of male victims of IPA. A more transparent legal redress process should be established to enable male victims to regain their trust in the legal process during IPA cases and encourage them to contact the police and courts about their victimization. Further studies might help understand how mandatory policies, directly and indirectly, affect male victims of IPA. This study should not be used as a justification or basis for the arrest of any alleged female aggressors without due process or taking resources from women’s abuse programs and DV homes, as suggested by Brooks et al. (2020). There have been concerns that opening up the experiences of male victims of IPA will lead to the shutting down of female victims. IPA has a negative physical and psychological impact on its victims, regardless of the gender involved, which makes it preferably a human than a gender issue. Shedding light on male victims, as well as female victims of IPA, can be used to improve the current state of services for victims and overall reduce the level of violence in the couples and families.

## Author’s Note

Eugene Emeka Dim is now affiliated with University of Toronto, Canada.

## Author Biographies

**Eugene Emeka Dim**, MA, is a PhD student in the Department of Sociology at the University of Toronto, Canada. His research generally focuses on intimate partner violence, gender studies, and political participation in Africa. His research draws on data from the Canadian General Social Survey on Victimization, Afrobarometer, and the Nigerian Demographic Health Survey.

**Alexandra Lysova**, PhD, is an assistant professor in the School of Criminology at Simon Fraser University in British Columbia, Canada. She has studied intimate partner violence, including violence against women and children, for over 15 years in Russia and currently in Canada. Recently, she has focused on men’s experiences of intimate partner violence, including victimization, help-seeking behaviour, and issues involving children. In her research, she draws on the results of the international focus groups among male victims in four English-speaking countries, Canadian General Social Survey on Victimization, and Homicide Survey. Dr. Lysova’s research has been supported by the prestigious foundations, including Trudeau, Fulbright, Carnegie and Humboldt foundations, and also U.S. Library of Congress and Max Planck Institute.
